# Telemedicine Service Use: A New Metric

**DOI:** 10.2196/jmir.1938

**Published:** 2012-12-19

**Authors:** Maurice Mars, Richard Scott

**Affiliations:** ^1^Department of TeleHealthNelson R Mandela School of MedicineUniversity of KwaZulu-NatalDurbanSouth Africa; ^2^Office of Global e-Health StrategyDepartment of Community Health Sciences, Faculty of MedicineUniversity of CalgaryCalgary, ABCanada

**Keywords:** telemedicine use, telemedicine metrics, telemedicine utilization, economics, evidence based, literature review

## Abstract

**Background:**

Policy makers and funding agencies require relevant information on current practices of the use of telemedicine infrastructure and services. Several metrics have been used to describe average use of telemedicine services. None are adequate.

**Objective:**

To identify and assess a new metric–consultations per site per week (C/S/W).

**Methods:**

To determine existing usage, all papers and abstracts published between January 2005 and December 2009 in the Journal of Telemedicine and Telecare and Telemedicine Journal and eHealth were reviewed. Pilot studies, research projects, services reporting less than one year’s data and teleradiology services were excluded.

**Results:**

In total, 210 reports of telemedicine services were identified, 77 of which provided sufficient data to calculate C/S/W. Average use was low, 1.8±3.5 (median 0.7) C/S/W, with 61% of services reporting less than 1 C/S/W and 71% reporting 2 or fewer C/S/W. Studies reporting on data from 2006 to 2009 showed less use (average 1.5±2.3; median 0.7 C/S/W) than earlier reports from 1996 to 2005 (1.7±2.5; median 0.7 C/S/W).

**Conclusions:**

The use of this new metric, C/S/W, is proposed as a standard measure of telemedicine service use. The generally low results opens debate about how well current clinical services are used.

## Introduction

Telemedicine, the delivery of healthcare services over distance using information and communication technologies, is slowly maturing. New telemedicine networks and programs are being implemented and established networks are growing. In recent years, some networks have reported tens of thousands of teleconsultations [1-3]. Telemedicine holds great promise for developing nations which, faced with large rural populations and few health professionals, are being encouraged to develop eHealth policies and strategies [4]. The African Union and the Pan American Health Organization (PAHO) have also both recently initiated work to develop collaborative-health strategy processes to harmonize continental activity. With limited budgets, poor existing telecommunication infrastructure, and expensive bandwidth [5], governments of developing countries seek to learn from the experiences of the developed world-hoping to bypass the pilot project cycle of telemedicine and implement sustainable projects. It is important that policy makers and planners have realistic expectations of telemedicine and set achievable goals. To do so, they require relevant information on the current norms of the use of telemedicine infrastructure and services.

What is the appropriate information? Different metrics have been used to report growth and use of telemedicine programs. In the first study to describe the growth in North American telemedicine activity between 1994 and 1999, Grigsby and Bennett reported the following metrics on an annual basis: the number of programs identified, the total number of teleconsultations across all programs, the average number of consultations per responding program, the total number of telemedicine sites reported and, the average number of sites per program. A steady annual increase was seen for all parameters [6].

While growth reported in this manner is useful, it does not describe the use of telemedicine at the level of the lowest common denominator, the referral site. When the number of programs and sites increases, there is likely to be an increase in the annual total and average number of consultations. This does not necessarily reflect activity at preexisting sites, which may be increasing, remaining constant, or even decreasing. A more relevant measure of telemedicine activity would be the number of teleconsultations per referral site per week. Using this metric on the data from Grigsby and Bennett’s study, which reported annual increases in all parameters, the number of consultations per site per week (C/S/W) fell from 1.07 in 1997 to 0.75 C/S/W in 1998, and rose slightly in the first quarter of 1998 to 0.95 C/S/W (Table 1). The low use of telemedicine when reported in this way is surprising and raises the question of what constitutes average telemedicine use now, over 10 years later? It also highlights the need for consistent and common metrics that better describe telemedicine use.

**Table 1:** Growth in North American telehealth activity, 1994-1999, excluding teleradiology services [6].

	1994	1995	1996	1997	1998	1999^a^
Number of programs	24	49		132	157	179
Number of consultations	2110	6138	21,732	41,740	52,223	74,828
Average consultations per program	88	125	253	316	428	608
Total number of facilities				747	1345	1521
Average facilities per program				8.3	10.3	11.3
C/S/W				1.1	0.8	1.0

^a^ The 1999 data are based on predictions based on the first quarter data.

The aim of this study was to determine reported telemedicine service usage, based on the new metric that measures C/S/W by reviewing all papers, abstracts, and letters published in 2 leading telemedicine-focused journals over the past 5 years.

## Methods

We reviewed all papers, abstracts, reports, and letters published between January 2005 and December 2009 in the 2 leading telemedicine journals, Telemedicine and e-Health and the Journal of Telemedicine and Telecare. Data from telemedicine programs were abstracted and confirmed by both authors. Data gathered included program duration, the number of teleconsultations, the number of referral sites, the nature of the telemedicine consultation (videoconferenced, email, Web, or telephony based), the unit or regional health service reporting the program and the country in which the program took place. Papers reporting services of less than one year’s duration and designated research or pilot projects were excluded. In keeping with Grigsby and Bennett’s paper, reports of radiological services were also excluded to enable direct comparison [6]. We have chosen not to include Diabetic Retinopathy screening services in the analysis of clinical consultative services, but to present them separately. Where the same service was reported more than once, only the most recently reported annualized data were recorded. Where papers report data on an annual basis, the serial data were also recorded separately to reflect change in service.

The average number of C/S/W was calculated by dividing the total number of consultations reported per program by the number of referral sites and then dividing this result by the duration of the program, expressed in weeks. The relative frequencies of services measured in C/S/W were calculated and categorized into 4 groups: those reporting 0-1, 1.1-2, 2.1-5, and more than 5.

Further analysis was made of the available serial data from programs, where available. As more recently reported services may show higher usage, studies that included only data gathered between 2006 and 2009 were compared with those that reported data gathered before 2006.

Data sets are reported as the mean and standard deviation, and the median is given when data are not normally distributed. Frequencies were compared using Fisher’s exact test. Means were compared using a Mann-Whitney test or the Kruskal-Wallis test when the data were not normally distributed. Alpha was set at 5%.

## Results

A total of 2510 papers, reports, letters, and conference abstracts were reviewed, in which 210 telemedicine services were reported in 187 papers and abstracts. Use was calculated as C/S/W using data from 49 papers and 36 abstracts, covering programs of 1 to 10 years’ duration, from 19 countries, with 7 international services (Multimedia Appendix 1). Of the 85 programs, 46 (54%) were in the US, 16 (19%) in the EU, 5 in Canada, and 4 in Australia. The average teleconsultations per week, number of referral sites, and C/S/W for clinical services and diabetic retinopathy screening services are summarized in Table 2.

The relative frequencies of use of clinical and diabetic retinopathy screening services are shown in Table 3.

There were 5 clinical services reporting more than 5 C/S/W. These included 2-single referral site programs in dermatology (5.7 C/S/W) [7] and intensive care (10.3 C/S/W) [8], a prison service (8.9 C/S/W) [9], a primary care service (11.2 C/S/W) [10], and an emergency room service (25.6 C/S/W)[3].

Serial data were available for 11 clinical programs. Average use increased by 0.13±0.37 C/S/W, ranging from −0.54 to 1.02 C/S/W. The number of C/S/W decreased in 2 of these programs due to an increase in the number of referral sites without a concomitant increase [11] or reduction in workload [12]. Only 3 programs reported annual data over several years. The first was a paediatric burn service, in which the C/S/W rose annually from 0.02 in 2001 to 0.08 in 2006 [13]. The second was a dermatology service [14], where C/S/W started at 0.8, maximized to 1.1, then fell to 1. The third was a neurology service [15], where C/S/W started at 2.6, rose to 4.4, and then fell to 3.6.

Pre-2006 data were reported in 35 papers with an average use of 1.7±2.5 C/S/W (range 0.03-11.2, median 0.7, and 95% CI:0.6-1.7) and 20 papers reported data from 2006–2009 with an average use 1.5±2.3 C/S/W (range 0.06-10.3, median 0.7, and CI:0.4-2.5). The relative frequencies are shown in Table 4.

There was no difference between the 2 groups for the average number of C/S/W, (*P* =.81). Comparison of the relative frequencies was made by consolidating the data and comparing the number of programs with 2 or fewer C/S/W with those that had 2 or more C/S/W as there were fewer than 5 programs reported in 5 of the 8 frequency ranges. No significant difference was found (*P* =.84).

The number of referral sites ranged from 1 to 48,707 sites in a telephony based service [16] (median 10). Differences in the number of referral sites were noted between the various telemedicine modalities but these were not statistically significant (*P* = .38): email (n=14, range 1-271, median 10.5), videoconferencing (n=45, range 1-700, median 8.5), Web (n=5, range 1–120, median 5.0), mixed modalities (n=4, range 1–390, median 154.0), telephone (n=2, range 896-48,707, median 24,801.5). Comparison of the mean C/S/W for videoconferencing, email and Web based services showed no significant difference (*P* = .14). Telephone (n=2) and modem services (n=5) were excluded from analysis as the sample sizes were too small.

**Table 2:** The number of consultations per week, the number of referral sites in the program, and the number of C/W/S expressed as the average (±standard deviation), median, minimum, and maximum for 77 clinical services and 8 diabetic retinopathy screening services.

			Consultations/week	Referral sites	C/S/W
**Clinical services (n=77)**					
	Average		107.9±345.4	690.0±5545.9	1.8±3.5
	Median		6.8	10.0	0.7
	Min		0.04	1	0.01
	Max		1923.1	48,707	25.6
**Diabetic retinopathy services (n=8)**					
		Average	403.7±776.3	50.6±84.2	39.0±62.3
		Median	168.7	3.5	11.5
		Min	3.1	1	1.75
		Max	2307.7	200	185.9

**Table 3:** The number of programs (n) and percentage frequency based on the 4 categories of use for clinical telemedicine and diabetic retinopathy screening services.

C/S/W category		≤1	1.1-2	2.1-5	>5
**Clinical services (n=77)**					
	n	47	8	17	5
	Frequency (%)	61	10	21	7
**Diabetic retinopathy services (n=8)**					
	n	0	1	1	6
	Frequency (%)	0	13	13	75

**Table 4:** The number of programs (n), percentage frequency, average (±SD), and median number of C/S/W for services reporting pre- and post-2006 data.

C/S/W			≤1	1.1-2	2.1-5	>5
**2006-2009 (n=20)**						
	n		14	2	3	1
	Frequency (%)		70	10	15	5
	Average C/S/W		0.5±0.3	1.3±0.2	3.1±0.3	10.3
	Median		0.5	1.3	3.0	
**1996-2005 (n=35)**						
		n	21	4	9	3
		Frequency (%)	60.0	11.4	20.0	8.6
		Average C/S/W	0.3±0.03	1.6±0.4	2.6±0.8	8.6±2.8
		Median	0.3	1.7	2.3	8.9

## Discussion

Using the new metric of C/S/W, average use of telemedicine sites as reported in the telemedicine literature is low, with 61% of telemedicine sites sending less than 1 case a week and 71% of sites sending less than 2 cases a week. The consistently limited use may indicate a relative ceiling that limits the number of cases that can or will be referred from a site, based on the practice population, incidence and prevalence of pathology, and the experience of the referring doctor.

When compared to Grigsby and Bennett’s 1997 to 1999 data, reporting an average of 0.9 C/S/W [6] we show a doubling of use, albeit it off a low base, to 1.8 C/S/W. No difference was seen between studies reporting data from 2006 onwards and pre-2006 data. Even the serial data extracted from programs show little change over time. In 2007, a large network of 700 referral sites reported 30,000 consultations a year, equivalent to 0.8 C/S/W [2]. In a presentation to the American Telemedicine Association Meeting in 2009, these figures had increased to 53,000 consultations a year and 1500 referral sites, but C/S/W had decreased marginally to 0.7. This exemplifies the need for a metric that reflects actual use.

Diabetic retinopathy screening services use the infrastructure more frequently (39 C/S/W) and some services are mobile. These services were reviewed separately as their inclusion would markedly skew the results of the other clinical services, in the same way that teleradiology services were excluded from Grigsby and Bennett’s review.

These results can only be taken as indicators of telemedicine use as there are limitations to this study. The review was of only 2 telemedicine journals, and large clinical telemedicine series may only have been reported in specific specialty journals. Also, large regional services may have reached a state of maturity and are no longer reported. Furthermore, aggregation of data over several years may mask changes in use, and changes in the number of referral sites in a service during the reporting period may skew the average use per site.

One of the problems inherent in many of the reports is that they focused on one clinical activity and did not report use of the same infrastructure for other activities such as other clinical services, videoconferenced education or administrative and research meetings. Additional difficulties included inconsistent terminology as to what is defined as a site or a consultation, and imprecision in presentation of dates (eg, saying the period was from ‘2002 to 2005’ could be interpreted as either 3 or 4 years, or 156 to 208 weeks; for this study, it was interpreted as 3 years), causing error in calculation of C/S/W. Over half (55%, 104/189) of the services identified in this study did not provide enough data to determine C/S/W.

Despite these shortcomings, the new metric of C/S/W provides a simple measure of telemedicine use. Our study shows that C/S/W is low, and this should be taken into account when planning new services. These data suggest that new infrastructure should be shared between clinical disciplines and used for non-clinical activities as well to provide efficiencies of scale.

We encourage consistent application of this new metric, and the reporting of adequate data by which to calculate it, including: explicit information about dates (year/month/day for the reporting period), the total number of sites within the network or service during the reporting period), differentiation of volumes or percentage of clinical, administrative, research, and education (CARE) activities, and the total number of consultations made during the reporting period to reflect clinical activity. We suggest that this metric is a useful way of evaluating use of telemedicine services.

## Multimedia Appendix 1

Summary of data and sources.

## Abbreviations

C/S/W: Consultations per site per week
EU: European Union
PAHO: Pan American Health Organization
