# Supplementary material for: Telemedicine Service Use: A New Metric
Source: J Med Internet Res. 2012 Dec 19;14(6):e178. doi: 10.2196/jmir.1938 (PMC3799556; doi:10.2196/jmir.1938)
Supplement: Supplementary file 1 [file jmir_v14i6e178_app1.pdf]

Mars M, Scott R
Telemedicine Service Use: A New Metric
J Med Internet Res 2012;14(6):e178
URL: http://www.jmir.org/2012/6/e178/
doi: 10.2196/jmir.1938

Mutlimedia Appendix 1
Summary of data and sources

Total Consults	Sites	Duration Wks	Cons/Site/ Week	Specialty	Country	Technology	Ref
14	3	312	0.01	Dermatology	USA	SF	1
6	2	104	0.03	Emergency	USA	VC	2
200	135	52	0.03	Psychiatry	Finland	VC	3
3,560	271	520	0.03	Mixed	China	Mixed	4
88,103	48,707	52	0.03	Cardiac - Teletriage	Israel	Tel	5
12,576	896	364	0.04	Cardiology	Italy	Tel	6
36	19	52	0.04	Child abuse	USA	email	7
10	2	104	0.05	Neonatology	USA	VC	2
1,000	65	320	0.05	Paediatric burns	Australia	VC	8
206	67	52	0.06	Mixed	Global	email	9
44	10	74	0.06	Paediatric Crit Care	USA	VC	10
100	8	156	0.08	Parkinson's	USA	VC	11
75	16	52	0.09	Surgery	USA	VC	12
819	75	104	0.11	Mixed	Australia	VC	13
132	3	416	0.11	Echo cardiology	Ireland	VC	14
1,672	212	52	0.15	Mixed	Canada	VC	15
505	29	108	0.16	Dermatology	Netherlands	e-Mail	16
767	30	134	0.19	Paediatric	International	VC	17
46	2	100	0.23	Cytology	India	e-Mail	18
165	12	52	0.26	Neurology Stroke	USA	VC	19
88	2	147	0.30	ER - stroke	Canada	VC	20
3,000	30	312	0.32	Mixed	USA	VC	21
66	1	195	0.34	Surgery	India	VC	22
1,500	80	52	0.36	Mixed	USA	VC	23
40	1	104	0.38	Cardiology	Serbia/ UK	VC	24
200	10	52	0.38	Paediatrics - Abuse	USA	VC	25
43	1	104	0.41	Paediatric ICU	USA	VC	26
1,458	14	247	0.42	Audiology	USA	SF	27
23	1	52	0.44	Trauma U/S	Canada	VC	28
1,684	68	52	0.48	Telemental Health	Canada	VC	29
500	20	52	0.48	Ante / neo natal care	USA	VC	30
3,000	120	52	0.48	Pathology	Japan	web	31
4,000	17	468	0.50	School / Psychiatry	USA	VC	32
132	5	52	0.51	Ophthalmology	International	web	33
606	9	130	0.52	Ped Mental Health	Australia	VC	34
217	3	130	0.56	Pathology	USA	SF	35
203	4	78	0.65	Psychiatry	Denmark	VC	36
311	3	156	0.66	Neurology	USA	VC	2
88	1	126	0.70	Paed Neuro-oncology	International	SF	37

Total Consults	Sites	Duration Wks	Cons/Site/ Week	Specialty	Country	Technology	Ref
18,250	84	312	0.70	Obstetrics	USA	Mixed	38
2,260	6	520	0.72	Psychiatry	USA	VC	39
3,800	100	52	0.73	Mixed	USA	VC	40
2,135	51	52	0.81	Prisons	USA	VC	41
30,000	700	52	0.82	Mixed	Canada	VC	42
900	19	52	0.91	Mixed	USA	VC	40
638	19	35	0.96	Mixed	USA	email	43
508	10	52	0.98	Mental Health	USA	VC & Tel	44
835	15	52	1.07	Trauma	Scotland	VC	45
2,000	16	104	1.20	Echocardiology	USA	VC	46
97	1	69	1.41	Primary care	Scotland	VC	47
7,500	20	260	1.44	Psychiatry	USA	VC	48
75	1	52	1.44	Stroke	Spain	VC	49
2,500	29	52	1.66	Mixed	USA	VC	50
214	1	119	1.80	Mixed	Cambodia	e-Mail	51
387	4	52	1.86	Psychiatry	USA	VC	52
343	1	156	2.20	Neuropathology	Austria	web	53
229	1	104	2.20	Mixed	USA	VC	54
2,009	12	74	2.26	Dermatology	Spain	SF	55
118	1	52	2.27	Ophthalmology	Australia	web	56
958	8	52	2.30	Mixed paediatric	USA	VC	57
385	1	156	2.47	Echocardiology	USA	Mixed	58
747	3	100	2.49	Dialysis / education	USA	VC	59
5,830	9	260	2.49	Cardiotocography	Italy	SF	60
26,777	31	342	2.53	Dermatology	USA	SF	61
297	1	104	2.86	Genetics	USA	VC	62
511	3	56	3.04	Psychiatry	USA	VC	63
700	4	52	3.37	Neurology Stroke	Germany	VC	64
108,159	224	139	3.47	ECG	Brazil	Mixed	65
1,804	2	260	3.47	Neurology	Ireland	VC	66
1,105	3	104	3.54	Psychiatry / other	USA	VC	67
60,000	275	52	4.20	Mixed	USA	VC	68
200,000	390	104	4.93	ECG	Chile	Mixed	69
451	1	79	5.71	Dermatology	USA	VC	70
52,000	112	52	8.93	Mixed - Prisons	USA	VC	71
535	1	52	10.29	Critical Care	USA	VC	72
1,164	2	52	11.19	Primary Care	USA	VC	73
80,000	12	260	25.64	Emergency	USA	VC	74

Total Consuls	Sites	Duration Wks	Cons/Site/ Week	Specialty	Country	Technology	Ref
15,678	172	52	1.75	Diabetic Retinopathy	USA	Web	[75]
318	1	104	3.06	Diabetic Retinopathy	International	SF	[76]
22,234	23	156	6.20	Diabetic Retinopathy	France	Web	[77]
120,000	200	52	11.54	Diabetic Retinopathy	USA	SF	[78]
1,800	1	156	11.54	Diabetic Retinopathy	USA	Web	[79]
12,701	6	60	35.28	Diabetic Retinopathy	USA	Web	[80]
2,962	1	52	56.96	Diabetic Retinopathy	Finland	CD	[81]
20,080	1	108	185.93	Diabetic Retinopathy	India	SF	[82]

Reference List

1 Sun A, Lanier R, Diven D. Teledermatology at the South Pole; 2009, p S-125.

2 Reyna M, Sable C, Conroy MB, Kushner DC. A comprehensive telemedicine solution for delivery of pedaitric care in suburban hospitals: factors influencing utilization of sub-specialty services; 2005, p 208.

3 Ohinmaa A, Roine R, Hailey D, Kuusimaki ML, Winblad I. The use of videoconferencing for mental health services in Finland. *J Telemed Telecare* **2008**;14:266-270.

4 Wang Z, Gu H. A review of telemedicine in China. *J Telemed Telecare* **2009**;15:23-27.

5 Roth A, Rogowski O, Yanay Y, Kehati M, Malov N, Golovner M. Teleconsultation for cardiac patients: a comparison between nurses and physicians: the SHL experience in Israel. *Telemed J E Health* **2006**;12:528-534.

6 Scalvini S, Mazzu M, Giordano A, Zanelli E, Piemontese C, Fedele F, Glisenti F. A review of seven years' telecardiology experience. *J Telemed Telecare* **2007**;13:50-52.

7 Blanchet KD. Innovative programs in telemedicine HealthBridge. *Telemed J E Health* **2009**;15:502-506.

8 Smith AC, Kimble R, O'Brien A, Mill J, Wootton R. A telepaediatric burns service and the potential travel savings for families living in regional Australia. *J Telemed Telecare* **2007**;13:76-79.

9 Wootton R. Telemedicine support for the developing world. *J Telemed Telecare* **2008**;14:109-114.

10 Heath B, Salerno R, Hopkins A, Caputo M. Rural peadiatric critical care telemedicine: summary of the Vermont experience; 2008, p 59.

11 Samii A, Ryan-Dykes P, Tsukuda RA, Zink C, Franks R, Nichol WP. Telemedicine for delivery of health care in Parkinson's disease. *J Telemed Telecare* **2006**;12:16-18.

12 Lavrentyev V, Seay A, Rafiq A, Justis D, Merrell RC. A surgical telemedicine clinic in a correctional setting. *Telemed J E Health* **2008**;14:385-388.

13 Dillon E, Loermans J, Davis D, Xu C. Evaluation of the Western Australian Department of Health telehealth project. *J Telemed Telecare* **2005**;11 Suppl 2:S19-S21.

14 McCrossan BA, Grant B, Morgan GJ, Sands AJ, Craig B, Casey FA. Diagnosis of congenital heart disease in neonates by videoconferencing: an eight-year experience. *J Telemed Telecare* **2008**;14:137-140.

15 Ohinma A, Scott R. A costing model for videoconferencing in Alberta. *J Telemed Telecare* **2006**;12:363-369.

16 Knol A, van den Akker TW, Damstra RJ, de Haan J. Teledermatology reduces the number of patient referrals to a dermatologist. *J Telemed Telecare* **2006**;12:75-78.

17 Mahnke C, Bervall E, Pinsker J, Person DA, Eble M. Pacific asynchronous telehealth: providing pediatric specialty consultation abroad; 2009, p S97.

18 Jialdasani R, Desai S, Gupta M, Kothari A, Deshpande R, Shet T, Ramadwar M, Kane S, Chinoy R. An analysis of 46 static telecytology cases over a period of two years. *J Telemed Telecare* **2006**;12:311-314.

19 Blanchet KD. Innovative programs in telemedicine: the University of Pittsburgh Medical Center (UPMC) Stroke Institute Telemedicine Program. *Telemed J E Health* **2008**;14:517-519.

20 Waite K, Silver F, Jaigobin C, Black S, Lee L, Murray B, Danyliuk P, Brown EM. Telestroke: a multi-site, emergency-based telemedicine service in Ontario. *J Telemed Telecare* **2006**;12:141-145.

21 Avram S, Ferrier S, Thomas S. Northern Sierra rural health network: connecting people to care; 2006, p 234.

22 Mishra A, Kapoor L, Mishra SK. Post-operative care through tele-follow up visits in patients undergoing thyroidectomy and parathyroidectomy in a resource-constrained environment. *J Telemed Telecare* **2009**;15:73-76.

23 Marcin JP, Nesbitt TS, Cole SL, Knuttel RM, Hilty DM, Prescott PT, Daschbach MM. Changes in diagnosis, treatment, and clinical improvement among patients receiving telemedicine consultations. *Telemed J E Health* **2005**;11:36-43.

24 Kosutic J, Rigby ML, Mijin D, Weatherburn G, Jowett V, Vukomanovic V, Rakic S, Markovic G. Low-bandwidth teleconsultations for patients with complex congenital heart diseases. *J Telemed Telecare* **2007**;13:113-118.

25 Jordan KL, Ramey E, Barnes MM, Ferrigno KJ, Mohr BA. University of Florida University of Florida,department of peditrics, child protection team: ligths action camera, telemedicine!; 2005, p 249.

26 Ellenby MS, Hoffman KG, Roe T, Burrell J. Oregon Health and Science Unicversity - Sacred Heart Medical Center pediatric critical care consult telemedicine program; 2009, p 722.

27 Kokesh J, Ferguson AS, Patricoski C, LeMaster B. Traveling an audiologist to provide otolaryngology care using store-and-forward telemedicine. *Telemed J E Health* **2009**;15:758-763.

28 Al Kadi A, Dyer D, Ball CG, McBeth PB, Hall R, Lan S, Gauthier C, Boyd J, Cusden J, Turner C, Hamilton DR, Kirkpatrick AW. User's perceptions of remote trauma telesonography. *J Telemed Telecare* **2009**;15:251-254.

29 Hailey D, Ohinma A, Roine R, Bulger T. Uptake of telemental health services in Alberta: a success, but not in all regions. *J Telemed Telecare* **2007**;13:42-44.

30 Hall-Barrow JC, MNP. Rural telemedicine: angels outreach and education; 2006, p 257.

31 Tofukuji I, Kanno K, Yamaguchi M, Ohyama N. Development of telepathology network based on on-demand vpn; 2005, p 263.

32 Nelson EL. Evolution of a school based program; 2008, p 50.

33 Kennedy C, Bowman R, Fariza N, Ackuaku E, Ntim-Amponsah C, Murdoch I. Audit of Web-based telemedicine in ophthalmology. *J Telemed Telecare* **2006**;12:88-91.

34 Smith AC, Stathis S, Randell A, Best D, Ryan VN, Bergwever E, Keegan F, Fraser E, Scuffham P, Wooton R. A cost-minimization analysis of a telepaediatric mental health service for patients in rural and remote Queensland. *J Telemed Telecare* **2007**;13:79-83.

35 Lopez AM, Barker G, Bhattacharyya AK, Scott K, Krupinski E, Richter L, Davenport J, Lazarus S, Kreykes L. Women's health through telemedicine: a panel discussion; 2009, p 69.

36 Mucic D. International telepsychiatry: a study of patient acceptability. *J Telemed Telecare* **2008**;14:241-243.

37 Qaddoumi I, Bouffet E. Supplementation of a successful pediatric neuro-oncology telemedicine-based twinning program by e-mails. *Telemed J E Health* **2009**;15:975-982.

38 Benton T, Manley M, Lowery CL, Ott R. Distant providers, close support: rural recruitment & retention through telemedicine; 2009, p S57.

39 Krupinski E, Barker GP, Lopez AM, Weinstein RS. 10- year analysis of telepsychiatry cases at an academic medical center; 2008, p 55.

40 Blanchet KD. Innovative programs in telemedicine: Great Plains Telehealth Resource and Assistance Center. *Telemed J E Health* **2008**;14:870-874.

41 Ellis DG, Mayrose J, Phelan M. Consultation times in emergency telemedicine using realtime videoconferencing. *J Telemed Telecare* **2006**;12:303-305.

42 Riesenbach R. Technical innovation in the creation of Canada's largest telemedicine network; 2007, p 169.

43 Pak H. Telethinking. *Telemed J E Health* **2007**;13:483-486.

44 Yellowlees P, Bourgeois JA, Hilty DM, Neufeld JD, Cobb HC. The e-mental health project at UC Davis- a consultation liaison primary care program with excellent clinical outcomes; 2006, p 211.

45 Miller DR, Alam K, Fraser S, Ferguson J. The delivery of a minor injuries telemedicine service by Emergency Nurse Practitioners. *J Telemed Telecare* **2008**;14:143-144.

46 Sable C, Reyna M, Hopkins P, Dixon RF, Sabouni S, Holbrook P. Evolution of pediatric telecarduiology practice: point to point wide area network; 2008, pp 75-76.

47 Webster K, Fraser S, Mair F, Ferguson J. Provision of telehealth to the Scottish Police College. *J Telemed Telecare* **2008**;14:160-162.

48 Cuyler RN. Telepsychiatry in rural outpatient geriatric care; 2008, p 78.

49 Pedragosa A, Alvarez-Sabin J, Molina CA, Sanclemente C, Martin MC, Alonso F, Ribo M. Impact of a telemedicine system on acute stroke care in a community hospital. *J Telemed Telecare* **2009**;15:260-263.

50 Antoniotti NM. Telethinking with Nina M. Antoniotti, R.N., M.B.A., Ph.D. Interview by Vicki Glaser. *Telemed J E Health* **2005**;11:517-521.

51 Heinzelmann PJ, Jacques G, Kvedar JC. Telemedicine by email in remote Cambodia. *J Telemed Telecare* **2005**;11 Suppl 2:S44-S47.

52 Myers KM, Valentine JM, Melzer SM. Child and adolescent telepsychiatry: utilization and satisfaction. *Telemed J E Health* **2008**;14:131-137.

53 Hutarew G, Schlicker HU, Idriceanu C, Strasser F, Dietze O. Four years experience with teleneuropathology. *J Telemed Telecare* **2006**;12:387-391.

54 Cole SL, Nesbitt TS. The financial impact of telemedicine; 2005, p 220.

55 Moreno-Ramirez D, Ferrandiz L, Ruiz-de-Casas A, Nieto-Garcia A, Moreno-Alvarez P, Galdeano R, Camacho FM. Economic evaluation of a store-and-forward teledermatology system for skin cancer patients. *J Telemed Telecare* **2009**;15:40-45.

56 Kumar S, Tay-Kearney ML, Chaves F, Constable IJ, Yogesan K. Remote ophthalmology services: cost comparison of telemedicine and alternative service delivery options. *J Telemed Telecare* **2006**;12:19-22.

57 Melzer SM, Valentine JM, Myers KM. Experience with pediatric telemedicine services in a regional network; 2008, p 74.

58 Huang T, Moon-Grady AJ, Traugott C, Marcin J. The availability of telecardiology consultations and transfer patterns from a remote neonatal intensive care unit. *J Telemed Telecare* **2008**;14:244-248.

59 Whitten P, Buis L. Use of telemedicine for haemodialysis: perceptions of patients and health-care providers, and clinical effects. *J Telemed Telecare* **2008**;14:75-78.

60 Di Lieto A, De Falco M, Campanile M, Torok M, Gabor S, Scaramellino M, Schiraldi P, Ciociola F. Regional and international prenatal telemedicine network for computerized antepartum cardiotocography. *Telemed J E Health* **2008**;14:49-54.

61 Burdick A, Berman B, Keri J, Alonso J, Wonderlich K, Lappan C, Avashia N. Teledermatology cases, University of Miami and Great Plains Regional Medical; 2009, p S-64.

62 Agan N, Franklin S, Hickey W, Perkins R, Jaramillo V, Wendel P, Lowery C, Orr R. Reflecting on rural practice: telemeidicine satellite clinic lessons learned; 2009, p S-57.

63 Chardos J, Agha Z, Fiedler J. No-show rate for TMH compared to face-to-face mental health; 2008, p 59.

64 Boeddicker A. European telehealth networks for oncology case discussions and stroke units. *J Telemed Telecare* **2006**;12:17-20.

65 Alkmim MB, Figueira RM, Ribeiro AL, Campos FE. Quality and cost improvements though telecardiology in Minas Gerais, Brazil; 2009, p S-29.

66 Bingham E, Patterson V. A telemedicine-enabled nurse-led epilepsy service is acceptable and sustainable. *J Telemed Telecare* **2007**;13:19-21.

67 Fox KC, Connor P, McCullers E, Waters T. Effect of a behavioural health and specialty care telemedicine programme on goal attainment for youths in juvenile detention. *J Telemed Telecare* **2008**;14:227-230.

68 Blanchet KD. Innovative programs in telemedicine. University of Texas Medical Branch at Galveston electronic health network. *Telemed J E Health* **2005**;11:116-123.

69 Fernandez FJ, Escobar E, Adriazola P, Aravena J. Technology considerations to reach 100% counrtywide tele-electrocardiographycoverage: the Chilean experience. *Telemed J E Health* **2007**;13:201.

70 Armstrong AW, Dorer DJ, Lugn NE, Kvedar JC. Economic evaluation of interactive teledermatology compared with conventional care. *Telemed J E Health* **2007**;13:91-99.

71 Hammack GG, Boltinghouse OW. Current status and operations of the UTMB digital medical services system; 2005, p 189.

72 Reynolds HN. Underserved does not necessarily mean rural. *Telemed J E Health* **2008**;14:103.

73 Hartshorn JC, Raimer BG. Primary care telemedicine: increased patient satisfaction and lower costs; 2005, p 247.

74 Galli R, Henderson K, Hall G. Telemergency - providing emergency care in a rural setting; 2009, p S64-S65.

75 Kim C, Zimmer-Galler I, Quinn K, Zeimer R. Utilization of a diabetic retinopathy assessment program in primary care; 2008, p 68.

76 Silva PS, Hock KM, Grossman MM, Cavallerano J, Sun JK, Aiello LM. Joslin Vision NMetwork - pediatric diabetes telemedicine eye care; 2009, p 719.

77 Erginay A, Chabois A, Gaudric A, Massin P. A screening program for diabetic retinopathy in the Paris area by using a telmedical network (OPHDIAT). *Telemed J E Health* **2008**;14:108.

78 Dayhoff R, Kuzmak P, Werfel J, Stuart F, El Hattah O. VA's nationwide telemedicine imging cpapbilities for cliniical specialties; 2009, p S-76-S-77.

79 Coll K, Birkmire-Peters DP, Pelletier BA, Buesell S. Joslin vision network: eye care visits following JVN imaging. *Telemed J E Health* **2005**;11:244.

80 Jackson-Moore D, Crammer R, Saxon G, Harper R. Trip (Teleretinal Imaging Program) in VHA VISN 16. *Telemed J E Health* **2008**;14:69.

81 Lemmetty R, Makela K. Mobile digital fundus screening of type 2 diabetes patients in the Finnish county of South-Ostrobothnia. *J Telemed Telecare* **2009**;15:68-72.

82 Bai VT, Murali V, Kim R, Srivatsa SK. Teleophthalmology-based rural eye care in India. *Telemed J E Health* **2007**;13:313-321.
